# Nanoencapsulation of crocin in double-layer emulsions for improved stability and enhanced efficacy against depression

**DOI:** 10.1371/journal.pone.0289425

**Published:** 2023-10-05

**Authors:** Zahra Nazari, Javad Asili

**Affiliations:** 1 Food Quality and Safety Research Group, Food Science and Technology Research Institute ACECR, Mashhad, Iran; 2 Department of Pharmacognosy, School of Pharmacy, Mashhad University of Medical Sciences, Mashhad, Iran; Shiraz University, ISLAMIC REPUBLIC OF IRAN

## Abstract

On average, depression affects 7.7% of the population aged 15 and older. One of the types of medicinal plants used to treat depression is saffron, which is uplifting and relieves sadness. Crocin as a bioactive compound is effective against depression, but it is sensitive to temperature, pH, and oxygen, and its efficiency decreases. Nanoencapsulation of crocin using double-layer emulsions is a method to increase the stability of this compound. Surfactant ratio (50% to 200%), stirrer speed (500 and 1000 rpm), and stirring time (45, 90, and 135 min) were used as variables of primary emulsion production. The optimum ratio of surfactant to aqueous phase was 100%, and in all microemulsions, viscosity increased with increasing surfactant ratio. A high-pressure homogenization method was used to make the secondary emulsion (double-layer emulsions), and soy protein concentrate (SPC), Gum Arabic (GA), and Pectin (P) at two levels of 5 and 10% were used to increase the stability. All treatments evaluated using Duncan’s test at a significance level of 5%. Double-layer emulsions stabilized with pectin showed the highest viscosity and the lowest release of crocin in simulated stomach and intestine conditions. The use of the cellular automata model to investigate the release of crocin showed that this model could simulate the effects of crocin concentration, polymer concentration, and the droplet size of double-layer emulsions with 93–99% confidence to predict the release in double-layer emulsions. Then the double-layer emulsions added to the chocolate, and the chocolate containing the double-layer emulsions compared with the control sample in terms of sensory properties and had a higher score than the control sample.

## Introduction

Depression affects 20% of the world’s population and is one of the ten main causes of death [1, 2]. Lithium salts, tricyclic antidepressants, selective serotonin inhibitors, monoamine oxidase inhibitors, etc. are used to treat depression [3, 4]. These drugs show their effects after several weeks to several months of use, and the symptoms of depression are completely resolved in only one third of the users. In addition, there is still a risk of a recurrence of the disease. Recently, typical antidepressants such as Bupropion, Nefazodone and Mirtazapine have become available for the treatment of depression. However, the rate of recovery is low and the risk of disease return is still high, so there is a need for compounds with greater efficacy and less side effects [5, 6].

Some plant extracts are effective sources of new drugs for the treatment of depression. Medicinal plants have already been used to treat various diseases, such as mental and psychological diseases, and have fewer side effects than synthetic and chemical drugs [5, 7, 8]. Some studies show that alcoholic and aqueous extracts of saffron have anti-depressant effects in rats [9, 10]. It has been determined that 94% of the total amount of crocin in saffron is in the form of glycosylated crocin and 6% is free crocin. Crocin has low stability and loses most of its activity after exposure to heat, oxygen, light, an acidic environment, and the presence of additives during food processing and storage [11]. The nano- and microencapsulation of crocin are two methods utilized to enhance its stability. Nanoemulsions are nano-sized (≤100 nm) colloidal systems formed by dispersing one liquid in another immiscible liquid using suitable surfactants. Surfactant is needed to produce crocin nanoemulsion. Surfactants with low HLB (hydrophob Lypophile balance) are used to prepare water-in-oil emulsions. Poly Glycerol Polyricinoleate (PGPR) is a food additive that is used as an emulsifier in the chocolate industry. Multiple emulsions are a group of structural emulsions that include small droplets of one phase embedded in larger droplets of another phase that are dispersed in a continuous phase, such as water in oil in water (W1/O/W2) and oil-in-water-in-oil (O1/W/O2) [12]. W1/O/W2 emulsions consist of an initial water-in-oil emulsion (W1/O) that is dispersed in another aqueous phase (W2). Double-layer emulsions are used as a vehicle for the encapsulation and delivery of hydrophilic bioactive compounds and drugs [13]. Several polymers and biopolymers are used for the synthesis of Double-layer emulsions. Soy protein concentrate (SPC) is prepared by removing soluble carbohydrate fractions as well as some flavor compounds from a defatted meal [14]. Gum Arabic (GA) is a plant-based exudate gum that consists of amino acids and polysaccharide units with poly glucuronic acid units. This poly anionic gum can directly absorb bioactive components. In addition, GA acts as a stabilizer as well as an emulsifier, which significantly increases the thermal stability of the nanoemulsion [15]. Pectin (P) is a polysaccharide composed of α-›1, 4 linked galacturonic acid with C-6 carboxyl groups esterified to a methyl group, and when the esterification is higher than 50%, it is considered high methoxy pectin, which can develop gels [16]. Macroscopic behavior is influenced by microscopic interactions. Cellular automata is a mathematical model that shows that complex systems can be simulated based on the interaction of their components with each other. Bertrand et al. (2007) used cellular automata to model drug release from erodible microspheres. Cellular automata were defined in three-dimensional conditions and with Moore’s law [17]. The different states were polymer, drug, space, and solvent. In the last few decades, chocolate has become one of the most popular foods worldwide [18]. Considering its high consumption, the fortifiication of chocolate with different bioactive compounds as a delivery agent seemed like a reasonable option. In addition, the knowledge gained from the study of Hadnađev et al. (2023) showed that the incorporation of encapsulated forms of commercial microencapsulated powder in dark chocolate was the best option in terms of product quality compared to other forms [19]. In this research, crocin double-layer emulsions was produced by microemulsification; after that, biopolymers such as SPC, GA, and pectin were used to control the release; then, cellular automation and experimental models of crocin release were compared. Finally, the physicochemical, rheological, and sensory characteristics of chocolate containing crocin double-layer emulsions were evaluated.

## Materials and methods

### Preparation of water-in-oil microemulsion

To prepare a water-in-oil microemulsion, lipophilic surfactant PGPR with HLB 1.5 was used. 10% w/w crocin solution was mixed with PGPR surfactant on a magnetic stirrer. The aqueous phase, containing surfactant, was added to the oil phase at 80% w/w. Then the resulting emulsion was mixed with constant rotation. Surfactant ratio (50% to 200%), stirring speed (500 and 1000 rpm), and stirring time (1, 2, and 3 hours) were used as variables for producing emulsions.

### Preparation of double-layer emulsions

10% solutions of pectin, GA and SPC were prepared by dissolving the samples in distilled water and keeping them overnight for complete hydration. 10% w/w water-in-oil microemulsion was added to a 90% w/w aqueous solution containing biopolymers and mixed for 5 minutes at 1000 rpm by ultrathorax. In order to make the final emulsion, the emulsion entered the high-pressure homogenizer from the high-speed homogenizer and was processed at a pressure of 10,000 in three cycles, and the samples were placed in glass tubes immediately after production.

### Droplet size analysis

Droplet sizes of microemulsion and double-layer emulsions were measured using the dynamic light scattering method (Scatteroscope, Korea).

### Viscosity

The viscosity of microemulsions and double-layer emulsions was measured using a viscometer (LVDV Pro II, Brookfield, USA) with a S34 spindle at speeds of 5–200 rpm. The flow behavior of double emulsions was investigated by fitting shear stress and shear rate data into power law, Herschel-Bulkelley, Bingham, and Casson models.

### Dynamic oscillatory test

The rheometer device with the geometrical shape of a cone and plate, a cone diameter of 50 mm, and a cone angle of 2 degrees was used for oscillation tests and checking viscoelastic properties [20]. Rheometer Physical MCR 301 (Anton Paar), with a conical plate geometry, a cone with a diameter of 50 mm, and a cone angle of 2 degrees, was used for dynamic tests and viscoelastic properties. Amplitude sweeps (0.1–100% strain) were performed at a constant frequency (1 Hz) to determine the linear viscoelastic region, where rheological properties are independent of stress or strain [21].

### In vitro release of crocin from double-layer emulsions

Crocin release experiments were studied in gastric (pH 1.2) and intestine (pH 6.8) solutions using the dialysis technique. The double emulsion was placed inside a dialysis bag (cellulose membrane, molecular weight cut-off 12.4 kDa, Sigma-Aldrich Co., St. Louis, MO). Moreover, it is immersed in a container containing 150 ml of 10 mM phosphate buffer solution (pH 7.4). The release system was kept at a temperature of 37°C in a magnetic stirrer (100 rpm). The bags were placed in the gastric buffer for 2 hours and then in the intestinal buffer for 6 hours. At specific times, the released crocin was measured with a spectrophotometer at a wavelength of 440 nm. Experiments were performed in triplicate for both samples [22].

### Diffusion coefficient of crocin

The Higuchi model was used to determine the release kinetics of double-layer emulsions [23].

### Modeling with cell automation

In this method, the types of positions are determined for the cells, and then the relationships between the cells are defined based on the characteristics of the emulsion and the crocin release process.

### Preparation of chocolate bars

Crocin double-layer emulsions were added to dark chocolate in a heated ball mill mixer (at 50°C) at a speed of 90 rpm for 30 minutes (15 mg crocin double-layer emulsions/10 g chocolate). Then this mixture was manually placed on the cold marble surface for tempering, poured into chocolate molds 5 cm long, 2.5 cm wide, and 0.2 cm high, and cooled at 4°C for 90 minutes. The samples were wrapped in aluminum foil and stored at 20°C.

### Color measurement

The color characteristics in terms of L*, a*, and b* were determined by Hunter Lab (color flex 45/0, USA).

### Texture analysis

The breaking force of chocolate bars was evaluated using a texture analyzer (TA Plus, Ametek, England) with a 3-point bending test and a 5-kg load cell at a speed of 2 mm/s. The test was performed in three repetitions at 20°C, 14 days after chocolate preparation.

### Physicochemical tests of chocolate

Physicochemical tests, including water activity, moisture, particle size, viscosity, and sensory evaluation of chocolate fortified with crocin double-layer emulsions, were evaluated.

### Statistical analysis

The effect of various variables, including the ratio of surfactant to water phase rate (SWR) (50 to 200%), stirring speed (500 and 1000 rpm), stirring time (45, 90, and 135 min), the type of biopolymer (SPC, GA,Pectin), and the concentration of biopolymers (5 and 10%), on the production of crocin double-layer emulsions were investigated. In addition, the effect of adding crocin double-layer emulsions antidepressant chocolate properties, were measured in three replications and compared with Duncan’s multi-range test using SPSS 22 software. Curve Expert Professional software (version 2.2) was used to model the experimental data of crocin flow behavior and release.

## Results and discussion

### Microemulsion production

#### PGPR concentration

To investigate the effect of PGPR concentration on the ability to produce microemulsion by the conservation method, SWR ratios from 50 to 200 were investigated. Other conditions were constant. The results showed (Table 1) that the smallest diameter was related to the ratio of surfactant to the aqueous phase of 100%, and with the increase of the ratio of surfactant to the aqueous phase, the droplet size increased and reached 56 and 115 nm in ratios of 150 and 200, respectively. In the research conducted by Saberi et al. (2013) on the production of oil-in-water microemulsions, it was shown that by increasing the ratio of surfactant to oil phase up to 100%, the droplet size decreased and then increased [12].

**Table 1:** Effect of SWR, stirring time, and particle size of microemulsions at standard conditions.

	SWR				Stirring speed (rpm)		Stirring time (min)		
	50	100	150	200	500	1000	45	90	135
**particle size (nm)**	11.18±0.22^a^	22.55±0.21^b^	56.23±0.43^c^	115.06±0.43^d^	11.40±0.32^a^	22.55±0.21^b^	9.32±0.13^a^	22.55±0.21^b^	53.06±0.01^c^

^*^Different letters in the same column indicate significant differences between treatments (P<0.05)

#### Stirring speed

The effect of stirring speed on the formation of microemulsions in standard conditions, i.e., when the ratio of surfactant to the aqueous phase is 100% and the time is 90 min, was evaluated at speeds of 500 and 1000 rpm. The results showed (Table 1) that at low speed, double-layer emulsions is not formed; in fact, there is no energy to separate the dispersed phase from the surfactant. The average size of the particles was about 11.40 nm, at 500 rpm, and when the speed increased to 1000 rpm, the droplet size was 22.55 nm. The increase in energy causes the agglomeration of the droplets before the movement of the surfactant in the continuous phase and its placement on the surface of the newly formed droplets [13].

#### Stirring time

To investigate the effect of stirring time on the characteristics of microemulsions, they were subjected to 45, 90, and 135 minutes under standard conditions, i.e., SWR 100% and speed of 1000 rpm. The results showed (Table 1) that with increasing time, the average droplet size increased from 9.32 to 45.04 and 53.06 nm. In another study, it was found that if the oil/surfactant combination were quickly added to the aqueous phase in the production of oil-in-water microemulsions, it would spontaneously form viscous masses of oil/surfactant/water [14].

#### The effect of surfactant concentration on the viscosity of microemulsions

**Fig 1:**
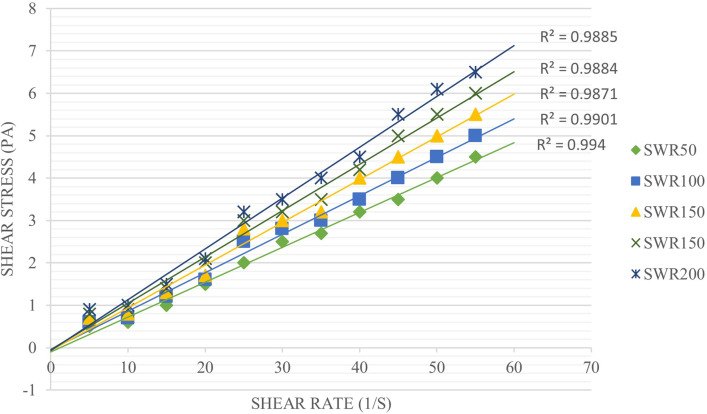
Flow chart of microemulsions.

The viscosity of emulsions has an effective role in the stability of emulsions, and with an increase in viscosity, the movement of dispersed phase droplets towards each other decreases and the accumulation of droplets is prevented. The results showed (Fig 1) that viscosity increased in all microemulsions with increasing SWR. This increase in viscosity can be due to the increase in free surfactant in the continuous phase, which leads to an increase in the viscosity of the emulsion [15].

### Double-layer emulsion production

#### Droplet size and stability of emulsions

As can be seen in Table 2, with increasing concentrations of SPC and GA, the droplet size decreased, but with increasing pectin concentration, the droplet size increased. The smallest diameter of double-layer emulsions was related to SPC, which decreased from 412 to 320 nm with increasing concentration from 5% to 10%. In the case of GA, the particle diameter decreased with increasing concentration, but for pectin, the particle size increased with increasing concentration. The droplet size of emulsions after the storage period showed that the diameter of all emulsions increased with storage time. GA 5% had the highest increase in droplet size, and pectin 5% had the lowest. The stability of the emulsion and the non-growth of globules over time are a function of the complete surface coverage and preventing the droplets from approaching each other. Also, studies have shown that the use of polymer compounds with a higher molecular weight leads to the formation of more stable double-layer emulsions [16].

**Table 2:** Droplet size average and dispersion of two-layer emulsions prepared from biopolymers.

	SPC5	SPC10	GA5	GA10	P5	P10
**Fresh emulsion**	412±3.33^a^	320±4.31^b^	645±4.35^c^	474±3.15^d^	1036±5.13^e^	1119±4.53^f^
**1** ^**th**^ **month**	498±4.36^a^	376±3.43^b^	754±4.75^c^	610±3.03^d^	1298±3.84^e^	1987±2.45^f^

^*^Different letters in the same column indicate significant differences between treatments (P<0.05)

#### Flow behavior

The evaluation of the viscosity of biopolymers at 5 and 10% levels showed that, at the shear rate range, all biopolymers showed thinning behavior at all levels. In all biopolymers, viscosity increased with increasing concentration, from 5 to 10%. The higher viscosity of pectin led to the formation of larger droplets and greater stability of double-layer emulsions in both concentrations (Table 3). The high viscosity of pectin prevented the droplets from getting closer to each other during the storage time and caused the emulsions stabilized with pectin to be more stable compared to the other two biopolymers.

**Table 3:** Biopolymers viscosity changes at 5 and 10% levels.

Shear rate (1/s)								
		10	20	30	40	50	60	70
Viscosity (mpa.s)	**SPC 5%**	7.33±0.12^a^	8.21±0.14^b^	8.94±0.23^b^	10.37±0.45^c^	10.67±0.36^c^	9.45±0.25^c^	8.76±0.23^b^
	**SPC 10%**	7.13±0.31^a^	7.33±0.24^a^	7.34±0.41^a^	6.73±0.12^a^	6.46±0.31^a^	5.91±0.07^a^	5.11±0.22^a^
	**GA5%**	9.06±0.13^c^	9.03±0.31^c^	9.13±0.32^c^	8.32±0.31^b^	8.45±0.34^b^	8.12±0.23^b^	8.33±0.21^b^
	**GA10%**	9.87±0.12^b^	10.13±0.32^d^	10.24±0.13^d^	11.65±0.21^d^	11.07±0.11^e^	11.11±0.32^d^	10.41±0.22^c^
	**P5%**	98.31±0.21^e^	96.21±0.12^f^	94.12±0.42^f^	93.01±0.33^f^	11.17±0.32^e^	93.23±0.32^f^	92.02±0.22^e^
	**P10%**	73.32±0.22^d^	74.43±0.21^e^	74.31±0.22^e^	74.08±0.21^e^	10.43±0.17^d^	73.12±0.21^e^	73.78±0.27^d^

^*^Different letters in the same column indicate significant differences between treatments (P<0.05)

The evaluation of the flow behavior (Table 4) based on the power law, Herschel-Balkley, Bingham, and Cason models shows that all biopolymers have the most compliance with the Herschel-Balkley model and then the power law. As can be seen, with the increase in concentration, the consistency coefficient (k) increases and the index (n) decreases, which indicates an increase in viscosity and approaching the dilute state (n < 1) with the increase in biopolymer concentration. The highest consistency coefficient and the lowest flow index were, respectively, pectin, GA and SPC. In the study of Benichou et al. (2007) on isolated whey powder in concentrations of 4 and 8%, it was shown that the flow behavior was Newtonian in both concentrations. The combination of xanthan gum with whey powder showed a pseudo-plastic behavior where, with the increase in gum concentration, the consistency coefficient (k) increased, and the flow index (n) decreased [17].

**Table 4:** Flow behavior of two-layer emulsion stabilized with biopolymers at the levels of 5 and 10%.

Emulsion	SPC		GA		P	
	5%	10%	5%	10%	5%	10%
Power law						
**K(Pa** ^**n**^ **)**	0.0032^d^	0.016^c^	0.009^cd^	0.013^cd^	0.059^b^	0.181^a^
**n**	0.991^a^	0.822^b^	0.917^ab^	0.814^ab^	0.816^ab^	0.811^ab^
**R** ^**2**^	99.8	98.8	99.6	99.1	99.5	98.1
Herschel bulkley						
**K(Pas** ^**n**^ **)**	0.001^c^	0.012^c^	0.007^c^	0.021^c^	0.043^b^	0.114^a^
**n**	0.933^a^	0.843^d^	0.914^ab^	0.867^cd^	0.901^abc^	0.856^bcd^
**τ**_**0**_ **(Pa)**	0.004^b^	0.222^a^	0.006^b^	0.018^b^	0.221^a^	0.220^a^
**R** ^**2**^	99.9	98.1	99.8	99.1	0.108	98.2
Bingham						
**K(Pas** ^**n**^ **)**	0.001^c^	0.002^c^	0.006^c^	0.008^b^	0.034^b^	0.201^a^
**τ**_**0**_ **(Pa)**	0.003^a^	0.232^a^	0.036^b^	0.038^b^	0.047^b^	0.114^bc^
**R** ^**2**^	99.9	98.1	99.7	99.2	99.4	78.9
Casson						
**K(Pas** ^**n**^ **)**	0.003^d^	0.047^dc^	0.005^c^	0.085^c^	0.401^b^	1.314^a^
**τ**_**0**_ **(Pa)**	0.012^b^	0.013^b^	0.007^b^	0.013^b^	0.014^b^	0.119^a^
**R** ^**2**^	92.2	98.3	93.1	99.6	99.9	97.8

^*^The components of the model for each biopolymer compared with each other in columns at a significance level of 0.05

#### Release of crocin

**Fig 2:**
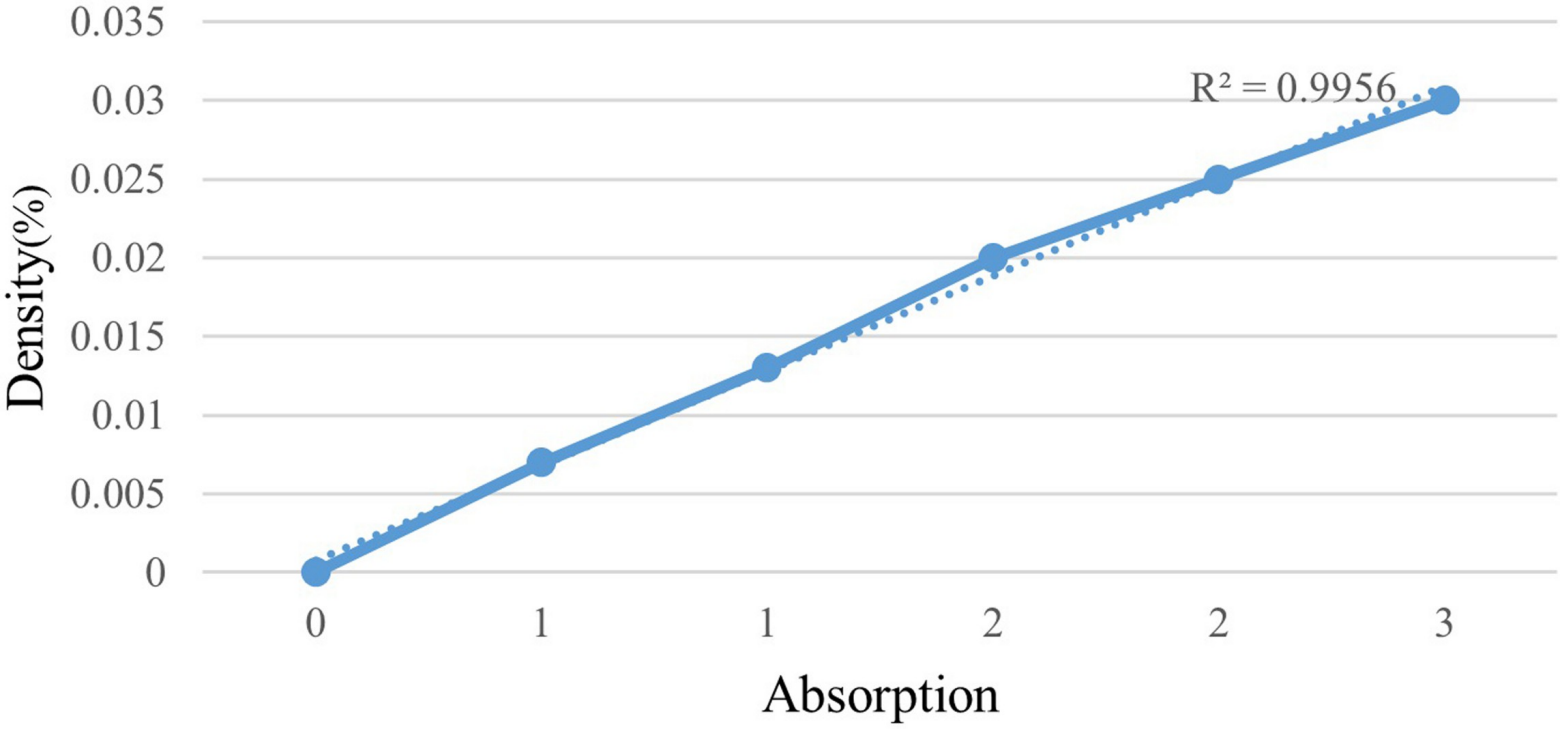
Crocin standard curve.

To evaluate the release of crocin during the storage time, the standard curve of crocin was determined based on the absorbance at 440 nm wavelength and in w/w concentrations of crocin (Fig 2). The detection coefficient (99.56%) showed that there is a high correlation between crocin concentration and absorption rate.

The release of crocin is shown in Table 5. The highest release of crocin was SPC, and the lowest was pectin. It seems that the macromolecule pectin is a factor in preventing its release. Mohammadi et al. (2016) produced double emulsions using whey powder and pectin and showed that adding pectin to the external aqueous phase and complexing it with whey powder reduces the release of phenolic compounds [18].

**Table 5:** Percentage of crocin release from double-layer emulsion stabilized with biopolymers at 5 and 10% levels.

Time (h)									
		1	2	3	4	5	6	7	8
**Crocin release**	**SPC 5%**	19.83±0.24^e^	38.15±0.34^f^	42.14±0.32^f^	53.87±0.31^f^	68.42±0.31^f^	75.18±0.14^f^	77.08±0.32^f^	78.43±0.41^f^
	**SPC 10%**	5.35±0.32^c^	15.76±0.25^d^	21.32±0.41^d^	28.94±0.22^d^	32.31±0.21^d^	43.21±0.32^d^	43.76±0.34^d^	47.35±0.31^d^
	**GA5%**	7.93±0.23^d^	20.78±0.13^e^	27.33±0.41^e^	39.74±0.42^e^	45.23±0.32^e^	51.76±0.12^e^	54.12±0.41^e^	56.22±0.22^e^
	**GA10%**	3.49±0.32^b^	10.76±0.16^c^	14.42±0.32^c^	21.21±0.06^c^	24.31±0.14^c^	32.76±0.17^c^	36.06±0.24^c^	39.31±0.21^c^
	**P5%**	3.97±0.24^b^	9.75±0.25^b^	13.43±0.16^b^	21.08±0.14^b^	23.66±0.08^b^	27.09±0.13^b^	28.14±0.13^b^	32.07±0.09^b^
	**P10%**	2.47±0.13^a^	6.77±0.15^a^	9.97±0.21^a^	12.06±0.08^a^	18.97±0.13^a^	19.98±0.15^a^	26.41±0.21^a^	26.85±0.08^a^

^*^Different letters in the same column indicate significant differences between treatments (P<0.05)

The methods of releasing compounds from polymer networks include diffusion, swelling of the polymer network, and decomposition of biopolymer materials. Because biopolymers are already hydrated, the mechanism of release in double-layer emulsions is diffusion. Breaking due to strong shearing force or due to the swelling of internal water droplets may lead to tearing, and it is possible to diffuse through narrow channels. The release component depends on the geometric shape of the pharmaceutical structure and the physical mechanism of release. The evaluation of crocin release using mathematical models in double-layer emulsions (Table 6) showed that the best model to explain the behavior of crocin release in the stomach is the Rigter-Peppas model, then the zero model. The best model in the intestine is the Peppas model and the first-order model in general. The high release rate, n, in the stomach showed that initially, due to the high concentration gradient of crocin, the release of the bioactive compound is independent of time and approaches the linear model.

**Table 6:** Crocin release models parameters in two-layer emulsions prepared with biopolymers in concentrations of 5 and 10%.

Condition	polymer	Zero order		First order		Higuchi		Rigter peppas		
		R	K	R	K	R	K	K	n	R
Stomach	SPC5	0.967	17.834	0.971	0.321	0.679	21.985	18.101	1.213	0.999
	SPC10	0.988	9.456	0.993	0.121	0.895	10.653	10.114	0.961	0.996
	GA5	0.991	10.108	0.995	0.126	0.798	12.985	12.235	1.112	0.997
	GA10	0.978	7.828	0.992	0.056	0.943	6.894	5.741	0.941	0.996
	P2.5	0.956	6.546	0.978	0.076	0.841	6.985	4.475	1.345	0.997
	P5	0.985	2.576	0.981	0.043	0.954	5.105	3.521	1.384	0.997
Intestine	SPC5	0.656	11.987	0.932	0.222	0.935	30.125	32.541	0.514	0.954
	SPC10	0.978	6.798	0.963	0.076	0.961	15.985	12.415	0.714	0.976
	GA5	0.735	7.678	0.945	0.123	0.95	19.175	19.021	0.455	0.976
	GA10	0.987	5.567	0.993	0.056	0.912	12.987	6.852	0.945	0.989
	P2.5	0.678	3.111	0.875	0.063	0.956	12.102	9.996	0.654	0.965
	P5	0.931	2.843	0.961	0.043	0.931	9.123	6.123	0.842	0.963
Total release	SPC5	0.876	11.675	0.993	0.253	0.964	27.991	23.123	0.714	0.971
	SPC10	0.951	7.124	0.988	0.087	0.974	16.112	10.213	0.721	0.994
	GA5	0.941	7.978	0.991	0.988	0.961	20.101	15.151	0.698	0.986
	GA10	0.996	5.634	0.987	0.995	0.925	11.914	7.201	0.698	0.997
	P2.5	0.943	4.451	0.988	0.976	0.952	10.945	6.989	0.881	0.979
	P5	0.978	3.967	0.978	0.991	0.912	7.985	4.987	0.854	0.984

#### Diffusion coefficient

**Fig 3:**
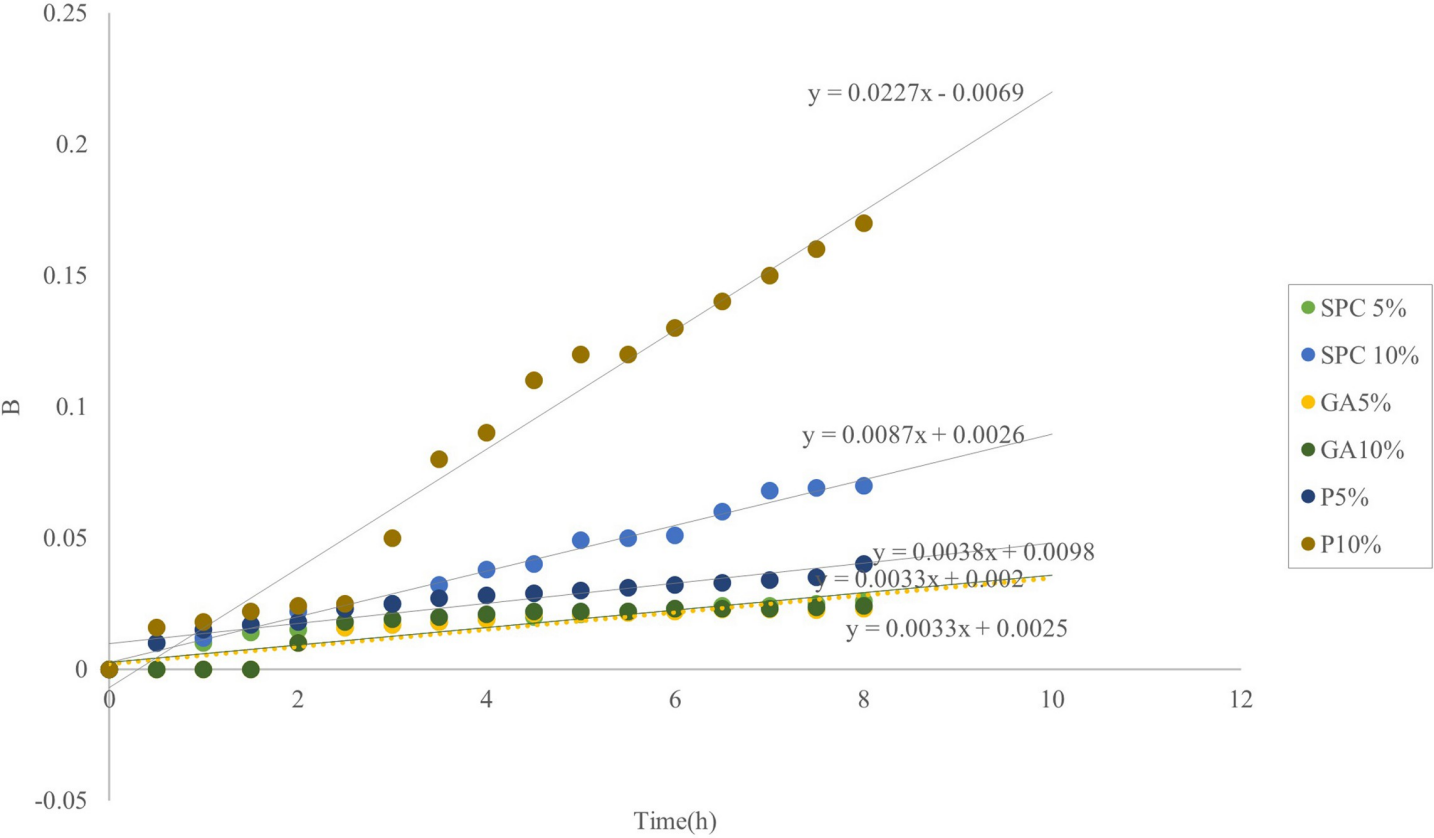
Relationship of release factor with to time for emulsions prepared with biopolymers in concentrations of 5 and 10%.

In the Higuchi model, the release factor, B, is directly related to time and inversely to concentration. To check the validity of the model, it is possible to check the relationship of the release factor to time or the inverse of the concentration. If the model is linear, the release mechanism is diffusion. The evaluation of the relationship between the release factors and time (Fig 3) showed a linear model between the factors with a high detection rate of 94%. This model represents the release based on the Higuchi model.

In pectin and GA emulsions, the diffusion coefficient decreased with increasing concentration from 5 to 10%, as well as with decreasing size and increasing viscosity (Table 7). In SPC emulsion, the diffusion coefficient increased due to the increase in droplet size along with the increase in concentration, despite the increase in viscosity. In these emulsions, increasing the diffusion coefficient due to the high viscosity did not cause a faster release of crocin.

**Table 7:** Effective diffusion coefficient of two-layer emulsions prepared with biopolymers in concentrations of 5 and 10%.

Emulsion	SPC5	SPC10	GA5	GA10	P5	P10
**D**_**e**_ **x 10**^**13**^**(cm**^**2**^**/h)**	7.67	1.24	8.04	1.24	6.87	6.87
**R** ^**2**^	0.97	0.98	0.097	0.98	0.98	0.98

#### Modeling by cellular automata

The effective factors in the modeling of crocin release are the state of cells, concentration of crocin, solubility in the oil phase and water phase, matrix size, and type of biopolymer. The release of crocin was evaluated in four different concentrations and in two solubility states for the oily phase. The results showed that increasing the concentration of the encapsulated compound increases the effective force for release, and the release amount of the bioactive compound increases at a constant time, which is similar to the results of other researchers in the release of various encapsulated compounds such as vitamins [19]. In the model designed with cellular automata, increasing the solubility increases the release of the bioactive compound. Bonnet et al. (2009) showed that the highest release of magnesium from double-layer emulsions was related to the medium-chain oil of myglyol and the lowest olein, and long-chain vegetable oils such as olive and rapeseed had a moderate release [20]. The size of the double emulsion affects the release rate of the bioactive compound by influencing the diffusion coefficient (Higuchi). In order to check this effect in the model, the dimensions of the matrix were checked in three modes: 100, 125, and 150 cells. The results showed that the release rate of the bioactive compound increased with a decrease in the size of the matrix. It was shown that changing the amount of biopolymer leads to changing the release rate of the bioactive compound from the internal aqueous phase to the external aqueous phase. Increasing the concentration of biopolymer, in addition to increasing the viscosity, increases the physical barrier on the external surface and reduces its release. To check the ability of the model to justify this effect, different levels of biopolymer are entered in the model, and it is observed that with the increase in biopolymer concentration, the release amount of the bioactive compound decreases.

To check the capability of the designed model using cellular automata, the simulated results were compared with the laboratory data. Because in all models, the concentration of crocin, oil, and internal water is constant, the effective parameters for comparing the results are the network size, the concentration of polymers in the external water, and the concentration of biopolymers around the oily phase. The obtained results showed that in the emulsions stabilized with SPC and GA, with increasing concentration, the diameter decreased, and the diffusion coefficient decreased, which indicated a lower amount of the bioactive compound released over time. In addition to changing the biopolymer concentration in the outer aqueous phase, the network size should also increase. In the double-layer emulsion stabilized with pectin, with the increase in biopolymer concentration, the droplet size is reduced, and the diffusion coefficient is reduced, which means that the dimensions of the network should be reduced to obtain the best output from the model. The ability of the model to simulate the release of crocin in these emulsions was more than 93% (Table 8).

**Table 8:** Release comparison with model results.

Emulsion	Crocin concentration	Polymer (%)	Compression	R^2^
**SPC5**	0.2	5	1	93
**SPC10**	0.2	10	1	99
**GA5**	0.2	5	2	97
**GA10**	0.2	10	2	99.3
**P5**	0.2	5	3	94
**P10**	0.2	10	3	98

### Chocolate tests

#### Physicochemical properties

The results of the analysis of variance showed that there is a significant difference (P<0.05) between chocolate containing double-layer emulsions in terms of moisture and water activity. As can be seen, by adding double-layer emulsions to chocolate, these factors increase. Also, there is no significant difference (P<0.05) between chocolate samples in terms of particle size index D90 (which means that 90% of the particles are smaller than this index) and D10 (which means that 10% of the particles are smaller than this index) (Table 9). As can be seen, with the addition of double-layer emulsions, the particle size did not increase significantly (P<0.05). The rheological characteristics and the final quality of chocolate are significantly (P<0.05) affected by the particle size distribution and compounds of chocolate, which play an important role in the sensory perception of this product. Coarse particles are effective in mouthfeel due to their sandy nature, but fine particles are more important due to their effect on the flow characteristics of chocolate [21]. Reducing the size of particles causes an increase in viscosity and yield stress. The size distribution of the particles is important because it can be optimized to achieve the desired rheological properties without any overall change in the chocolate formulation. If the maximum size of D90 is between 20 and 33 μm and the minimum size of D10 particles is less than 6 μm, the desired rheological properties will be achieved with the best mouthfeel. D90 greater than 35 μm creates a sandy mouthfeel, which leads to a decrease in overall acceptance by the consumer. As shown in Table 9, the highest viscosity and hardness in solid state (hardness1) and melted state (hardness2) are related to the fortified treatment, which can be due to the increase in humidity. Aeschlimann & Beckett (2007) reported that moisture (even in very low amounts) increases the hardness of chocolate [22]. The results of the analysis of variance showed that there is a significant difference between chocolate in terms of the a, b, and L factors (p < 0.05). As can be seen, by adding double-layer emulsions to chocolate, L* decreases and a^*^ and b^*^ increase.

**Table 9:** Comparison of mean physicochemical properties of crocin fortified chocolate.

		Control	Fortified
**Water activity**		0.30±0.15^a^	0.33±0.13^b^
**Moisture (%)**		0.26±0.32^a^	0.69±0.24^b^
**Viscosity (mpa)**		1043.54±0.50^a^	1098.43±0.21^a^
**Hardness1 (N)**		18.98±0.22^a^	21.75±0.13^b^
**Hardness2 (N)**		10.08±0.14^a^	12.43±0.24^b^
**Particle size (μ)**	**D90 (20–33)**	18.32±0.31^a^	18.87±0.24^a^
	**D10 < 6**	1.22±0.14^a^	1.12±0.16^a^
**color**	**L**	19.52±0.25^a^	19.04±0.13^b^
	**a**	10.53±0.33^a^	10.99±0.07^b^
	**b**	15.76±0.13^a^	16.14±0.34^b^

^*^Different letters in the same column indicate significant differences between treatments (P<0.05)

#### Sensory evaluation

**Fig 4:**
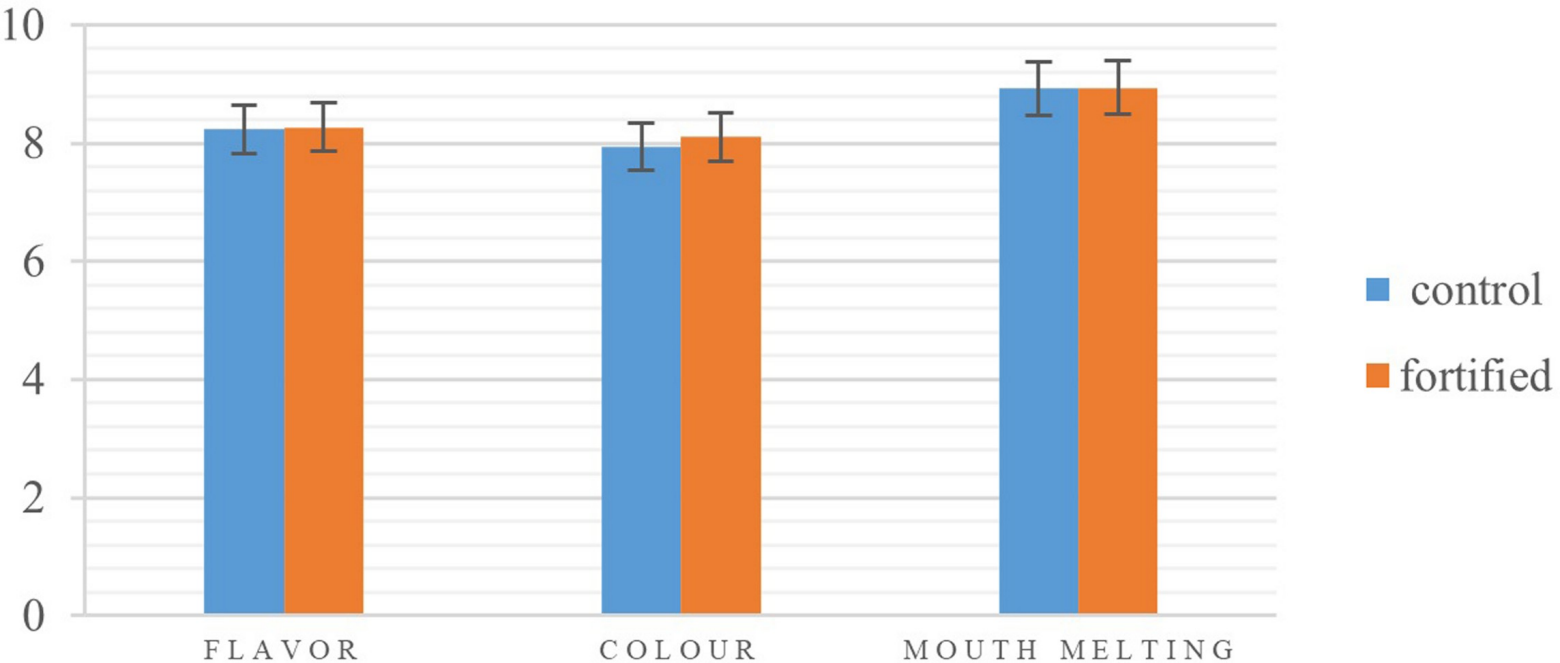
Effect of crocin double-layer emulsions on sensory properties of chocolate.

Sensory evaluation was done by the hedonic method (9 points), and chocolate samples were evaluated in terms of flavor, color intensity, and mouth melting. The fortified treatment had the highest flavor score (Fig 4). In terms of oral melting, there was no significant difference between the treatments. In terms of color intensity, the highest score was given to the fortified treatment.

## Conclusion

Crocin has high solubility in water, and its antidepressant effects have been proven. The evaluation of the release of double-layer emulsions in the simulation environment of the stomach and intestine showed that the lowest release is related to samples prepared with pectin biopolymer and the highest is related to SPC. The results showed that with increasing concentration, the diffusion coefficient (k) decreased. Modeling the release of crocin from double-layer emulsions showed that the designed model is able to show the differences in the concentration of the encapsulated compound, the dimensions of the network, and the solubility of the compound in the oil and aqueous phases, as well as the density of the polymer at the secondary interface. The conformity of the model with the laboratory results shows that the algorithm designed for the model is capable of simulating the release of crocin with a high accuracy of 93–99%, depending on the type of double-layer emulsion. The results of moisture, water activity, and hardness of chocolates showed that the fortified chocolates had higher hardness, water activity, and moisture than the control. The color evaluation showed that the fortified samples had less L* and more a^*^ and b^*^ than the control sample. Comparison of particle size and viscosity of control and fortified samples did not show a significant difference (P<0.05). The sensory evaluation showed that the fortified samples scored significantly (P<0.05) higher than the control. The results of this research showed that cellular automata could be used to model drug release instead of many treatments.

## Supporting information

S1 Data (XLSX)

## References

[1] Zhang F, Zhu X, Yu P, Sheng T, Wang Y, Ye Y. Crocin ameliorates depressive-like behaviors induced by chronic restraint stress via the NAMPT-NAD+-SIRT1 pathway in mice. Neurochemistry International. 2022;157:105343. doi: 10.1016/j.neuint.2022.105343 35490894

[2] Ghalandari-Shamami M, Nourizade S, Barati M, Yousefi B, Pashayi M, Ali Vafaei A, et al. Exercise and crocin prevent adolescent-stress induced impairment of spatial navigation and dendritic retraction in the hippocampal CA3 area in adult male rats. Brain Research. 2021;1754:147274. doi: 10.1016/j.brainres.2020.147274 33422526

[3] Tao W, Ruan J, Wu R, Zhao M, Zhao T, Qi M, et al. A natural carotenoid crocin exerts antidepressant action by promoting adult hippocampal neurogenesis through Wnt/β-catenin signaling. Journal of Advanced Research. 2022. doi: 10.1016/j.jare.2022.02.015. PMC9811320 36585110

[4] Scuto M, Modafferi S, Rampulla F, Zimbone V, Tomasello M, Spano’ S, et al. Redox modulation of stress resilience by Crocus sativus L. for potential neuroprotective and anti-neuroinflammatory applications in brain disorders: From molecular basis to therapy. Mechanisms of Ageing and Development. 2022;205:111686. doi: 10.1016/j.mad.2022.111686 35609733

[5] Xiao Q, Shu R, Wu C, Tong Y, Xiong Z, Zhou J, et al. Crocin-I alleviates the depression-like behaviors probably via modulating "microbiota-gut-brain" axis in mice exposed to chronic restraint stress. J Affect Disord. 2020;276:476–86. Epub 2020/09/03. doi: 10.1016/j.jad.2020.07.041 . 32871679

[6] Dai W, Feng K, Sun X, Xu L, Wu S, Rahmand K, et al. Natural products for the treatment of stress-induced depression: Pharmacology, mechanism and traditional use. Journal of Ethnopharmacology. 2022;285:114692. doi: 10.1016/j.jep.2021.114692 34742864

[7] Jin X, Guo J-L, Wang L, Zhong X, Yao W-F, Gao H, et al. Natural products as pharmacological modulators of mitochondrial dysfunctions for the treatments of Alzheimer’s disease: A comprehensive review. European Journal of Medicinal Chemistry. 2021;218:113401. doi: 10.1016/j.ejmech.2021.113401 33831779

[8] Abbaszade-Cheragheali A, Beheshti F, Kakhki S, Khatibi SR, Dehnokhalaji F, Akbari E, et al. Crocin, the main active saffron (Crocus sativus L.) constituent, as a potential candidate to prevent anxiety and depressive-like behaviors induced by unpredictable chronic mild stress. Neuroscience Letters. 2022;791:136912. doi: 10.1016/j.neulet.2022.136912 36244518

[9] Xie Y, He Q, Chen H, Lin Z, Xu Y, Yang C. Crocin ameliorates chronic obstructive pulmonary disease-induced depression via PI3K/Akt mediated suppression of inflammation. Eur J Pharmacol. 2019;862:172640. Epub 2019/09/07. doi: 10.1016/j.ejphar.2019.172640 . 31491407

[10] Fan H, Fu G, Feng S, He X, Cai W, Wan Y. Fabrication of casein-crocin nanocomplexes: Interaction mechanism, impact on stability and bioavailability of crocin. Food Hydrocolloids. 2023;136:108279. doi: 10.1016/j.foodhyd.2022.108279.

[11] Mir TuG, Wani AK, Singh J, Shukla S. Therapeutic application and toxicity associated with Crocus sativus (saffron) and its phytochemicals. Pharmacological Research—Modern Chinese Medicine. 2022;4:100136. doi: 10.1016/j.prmcm.2022.100136 .

[12] Saberi AH, Fang Y, McClements DJ. Fabrication of vitamin E-enriched nanoemulsions: factors affecting particle size using spontaneous emulsification. Journal of colloid and interface science. 2013;391:95–102. Epub 2012/11/03. doi: 10.1016/j.jcis.2012.08.069 . 23116862

[13] Jafari SM, Assadpoor E, He Y, Bhandari B. Re-coalescence of emulsion droplets during high-energy emulsification. Food Hydrocolloids. 2008;22(7):1191–202. doi: 10.1016/j.foodhyd.2007.09.006 .

[14] Yang Y, Marshall-Breton C, Leser ME, Sher AA, McClements DJ. Fabrication of ultrafine edible emulsions: Comparison of high-energy and low-energy homogenization methods. Food Hydrocolloids. 2012;29(2):398–406. doi: 10.1016/j.foodhyd.2012.04.009.

[15] An Y, Yan X, Li B, Li Y. Microencapsulation of capsanthin by self-emulsifying nanoemulsions and stability evaluation. European Food Research and Technology. 2014;239(6):1077–85. doi: 10.1007/s00217-014-2328-3

[16] Li C, Huang Q, Ma S, Ji C. An Experimental Study on the Viscosity of Water-in-Oil Emulsions. Journal of Dispersion Science and Technology. 2016;37(3):305–16. doi: 10.1080/01932691.2014.994218

[17] Benichou A, Aserin A, Lutz R, Garti N. Formation and characterization of amphiphilic conjugates of whey protein isolate (WPI)/xanthan to improve surface activity. Food Hydrocolloids. 2007;21:379–91. doi: 10.1016/j.foodhyd.2006.04.013

[18] Mohammadi A, Jafari SM, Assadpour E, Faridi Esfanjani A. Nano-encapsulation of olive leaf phenolic compounds through WPC-pectin complexes and evaluating their release rate. Int J Biol Macromol. 2016;82:816–22. Epub 2015/10/16. doi: 10.1016/j.ijbiomac.2015.10.025 . 26459167

[19] Hadnađev M., et al. (2023). "Fortification of chocolate with microencapsulated fish oil: Effect of protein wall material on physicochemical properties of microcapsules and chocolate matrix." Food Chem X 17: 100583. doi: 10.1016/j.fochx.2023.100583 36845485 PMC9945424

[20] Bonnet M, Cansell M, Berkaoui A, Ropers MH, Anton M, Leal-Calderon F. Release rate profiles of magnesium from multiple W/O/W emulsions. Food Hydrocolloids. 2009;23(1):92–101. doi: 10.1016/j.foodhyd.2007.11.016.

[21] Wells MA. Industrial chocolate manufacture and use (2nd edn): edited by S.T. Beckett, Blackie, 1994. £79.00 (xviii + 408 pages) ISBN 0 7514 0012 2. Trends in Food Science & Technology. 1994;5(11):375–6. doi: 10.1016/0924-2244(94)90218-6.

[22] Aeschlimann JM, Beckett S. International inter-laboratory trials to determine the factors affecting the measurement of chocolate viscosity. Journal of Texture Studies. 2007;31:541–76. doi: 10.1111/j.1745-4603.2000.tb01019.x

[23] Dukhin S. S. & Labib M. E. Theory of effective drug release from medical implants based on the Higuchi model and physico-chemical hydrodynamics. Colloids Surf A Physicochem Eng Asp 409, 10–20 (2012) doi: 10.1016/j.colsurfa.2012.04.040 24155569 PMC3804347

